# Retraction: Investigating Pollen and Gene Flow of WYMV-Resistant Transgenic Wheat N12-1 Using a Dwarf Male-Sterile Line as the Pollen Receptor

**DOI:** 10.1371/journal.pone.0265375

**Published:** 2022-03-09

**Authors:** The PLOS ONE Editors

Following the publication of this article [1], concerns were raised regarding the results presented in Fig 2. Specifically, repetitive patterns were identified in the background of lanes 2–9.

This concern was discussed with the first author and corresponding author, and raw image data underlying the figure in question were requested.

The authors commented that the similar areas in the background of Fig 2 may have been the result of image editing errors. The authors stated that the raw image data underlying the published figure is no longer available. They provided alternative image data which was described as PCR detection of the *Nib8* transgene in seedling samples. However, in the absence of the original data supporting the published figure, the journal does not consider that alternative experiment data are sufficient to resolve the image concerns.

In addition, the Data Availability statement for this article reads “All relevant data are within the paper and its Supporting Information files.” As the raw image data underlying Fig 2 are not available, the article does not comply with *PLOS ONE*’s Data Availability policy that was in place at the time of the article’s submission.

In the absence of the raw data, the concerns regarding the integrity of the image data in Fig 2 remain unresolved. Therefore, the *PLOS ONE* Editors retract this article.

SD agreed with the retraction. YL, CY, ZZ, MC and CW either did not respond directly or could not be reached.
